# sRage plasma level in association with multiple sclerosis risk in Slovak population

**DOI:** 10.1186/2193-1801-4-S1-P10

**Published:** 2015-06-12

**Authors:** Dusan Dobrota, Daniel Cierny, Jozef Michalik, Egon Kurca, Jan Lehotsky

**Affiliations:** Department of Medical Biochemistry, Comenius University in Bratislava, Jessenius Faculty of Medicine in Martin, Slovakia; Clinic of Neurology, University Hospital and Jessenius Faculty of Medicine in Martin, Slovakia

**Keywords:** multiple sclerosis, sRAGE, ELISA

Receptor for advanced glycation end products (RAGE) triggers an intracellular signalling pathways of various proinflammatory ligands. Solubile form of RAGE (sRAGE) in human plasma can hinder the function of RAGE and can have the role in the pathogenesis of inflammatory diseases. In patients with multiple sclerosis (MS), altered level of sRAGE in plasma and cerebrospinal fluid has been found. In our study, we tried to identify possible association of sRAGE serum level with MS. Serum levels of sRAGE were detected by ELISA in 44 MS patients (22 patients with rapid progression of MS and 22 patients with slow progression of MS according to the MSSS score) and 32 healthy control subjects. We found significantly increased level of sRAGE in serum of MS patients when compared to controls. No significant differences in serum level of sRAGE where found between rapidly progressing and slow progressing subgroup of MS patients. Our results suggest for the role of sRAGE in MS ethiopathogenesis, but we did not find any association of sRAGE in serum with the rate of MS disability progression.

